# Regulation of dynamic BACE1 trafficking in neurons

**DOI:** 10.1186/1750-1326-8-S1-O8

**Published:** 2013-09-13

**Authors:** Virginie Buggia-Prévot, Celia G Fernandez, Sean Riordan, Kulandaivelu S Vetrivel, Vinod Udayar, Aureliane Elie, Margaret Lefkow, Jelita Roseman, Xavier Meckler, Sohinee Bhattacharyya, Manju George, Jack Waters, Vytautas P Bindokas, Angèle T Parent, Lawrence Rajendran, Hamid Band, Robert Vassar, Gopal Thinakaran

**Affiliations:** 1 Departments of Neurobiology, Neurology, and Pathology, The University of Chicago, Chicago, IL 60637, USA; 2 Department of Pharmacological and Physiological Sciences, The University of Chicago, Chicago, IL 60637, USA; 3 Committee on Neurobiology, The University of Chicago, Chicago, IL 60637, USA; 4 Department of Cell and Molecular Biology, Feinberg School of Medicine, Northwestern University, Chicago, IL 60611, USA; 5 Department of Physiology, Feinberg School of Medicine, Northwestern University, Chicago, IL 60611, USA; 6 Systems and Cell Biology of Neurodegeneration, Division of Psychiatry Research, University of Zurich, 8008 Zurich, Switzerland; 7 Eppley Institute for Research in Cancer and Allied Diseases and Eppley Cancer Center, University of Nebraska Medical Center, Omaha, NE 68198, USA

## 

Proteolytic processing of amyloid precursor protein (APP) by β-site APP cleaving enzyme 1 (BACE1) and γ-secretase generates Aβ peptides. APP is constitutively trafficked through the secretory and endocytic pathways in cultured cells and neurons. The identities of cellular organelles and sorting pathways involved in amyloidogenic processing of APP have been extensively investigated. Although a consensus has not yet emerged, there is a general agreement from biochemical and genetic studies on the importance of endocytic APP trafficking for Aβ production. In neurons, APP is trafficked anterogradely along peripheral and central axons, and proteolytically processed during transit. Recent *in vivo* studies estimated that ~70% of Aβ released in the brain requires ongoing endocytosis, and synaptic activity regulates the vast majority of this endocytosis-dependent Aβ. BACE1 cleavage is thought to be the rate-limiting step in amyloidogenic processing of APP. Little, however, is known about endocytic BACE1 sorting and dynamic transport in neurons. We investigated BACE1 trafficking in cultured hippocampal neurons using live-cell imaging and selective labeling. This approach revealed dynamic neuronal transport of internalized BACE1 in dendrites and axons. BACE1 was transported in vesicles that were positive for markers of recycling endosomes. Dominant-negative expression and siRNA knock-down of proteins involved in endocytic transit revealed that BACE1 is dynamically transported in recycling endosomes and this process significantly contributes to amyloidogenic APP processing. Our results suggest that BACE1 trafficking in neuronal recycling endosomes is likely relevant for presynaptic Aβ production and contributes to Alzheimer’s disease pathogenesis.

